# Cardiomyocyte Proliferation in Embryonic Zebrafish Hearts Induced by Novel Small-Molecule Compounds

**DOI:** 10.51894/001c.163955

**Published:** 2026-08-01

**Authors:** Benjamin Xu, Daniela T. Fuller, Dalila Livadic, Aaron H. Wasserman, Charles H. Hong

**Affiliations:** 1 College of Osteopathic Medicine, Michigan State University

## 02

### BACKGROUND

Despite significant advances in preventive, pharmacologic, and surgical therapies, cardiovascular disease remains the leading cause of morbidity and mortality worldwide. In mammals, cardiomyocyte loss is largely permanent, and effective regeneration of cardiomyocytes after injury remains elusive. In contrast, zebrafish possess a unique ability to regenerate the heart after injury, making them an ideal model organism for studying the effects of regenerative therapeutics. LRN9 and DF6 are two novel small-molecule compounds that target the WNT/β-catenin signaling pathway, a pathway known to regulate cardiomyocyte proliferation.

### OBJECTIVES

Previous studies demonstrated that LRN9 and DF6 increase ventricular size in 2D human induced pluripotent stem cell (iPSC)-derived cardiomyocytes and 3D cardiac organoids. We hypothesize that LRN9 and DF6 would induce cardiomyocyte proliferation in vivo in zebrafish embryos. Additionally, we aimed to determine whether any observed increase in ventricular size was driven by cardiomyocyte hypertrophy, hyperplasia, or both.

### METHODS

Zebrafish embryos were collected and maintained in standard embryo media. Experimental groups were treated with dimethyl sulfoxide (DMSO) as a vehicle control, LRN9, or DF6. At 3 days post-fertilization, embryos were harvested and ventricular morphology was assessed using microscopy. Ventricular area and length were quantified using image analysis software. A cardiomyocyte nuclei–specific fluorescent dye was injected into zebrafish hearts prior to microscopy, enabling total cardiomyocyte nuclei quantification.

### RESULTS

Zebrafish embryos exposed to LRN9 and DF6 exhibited significant increases in both ventricular area and length compared to controls. LRN9 and DF6 treatment resulted in 1.2-fold and 1.3-fold increases in ventricular length and area, respectively. 1.4-fold and 1.6-fold increases in total cardiomyocyte nuclei counts were seen in LRN9- and DF6-treated embryos, respectively, indicating that ventricular enlargement was driven primarily by hyperplasia. No reductions in embryo viability or gross developmental abnormalities were observed in treated groups.

### CONCLUSIONS

These findings demonstrate that LRN9 and DF6 induce cardiomyocyte proliferation in vivo in zebrafish embryos through hyperplastic cardiac growth. Together with prior evidence of pro-proliferative effects in human iPSC-derived cardiomyocytes and cardiac organoids, these results position LRN9 and DF6 as promising candidates for further investigation as regenerative therapies for cardiomyocyte loss and heart failure.

